# Maximal functional capacity in subjects with isolated left bundle branch block: A pilot study

**DOI:** 10.1002/clc.23977

**Published:** 2023-02-08

**Authors:** Patricia Palau, Jose Mendez, Enrique Santas, Julio Núñez, Laura López, Antonio Briatore, Eloy Domínguez

**Affiliations:** ^1^ Cardiology Department, Hospital Clínico Universitario, INCLIVA Universitat de València Valencia Spain; ^2^ Cardiology Department IMED Elche Alicante Spain; ^3^ CIBER Cardiovascular Madrid Spain; ^4^ Facultad de Fisioterapia Universitat de València Valencia Spain; ^5^ Fisabio Universitat Jaume I Castellón Spain

**Keywords:** cardiopulmonary exercise testing, left bundle branch block, maximal functional capacity

## Abstract

**Background:**

Left bundle branch block (LBBB) has been commonly associated with adverse cardiovascular (CV) events, but the effect of an isolated LBBB on maximal functional capacity is not well characterized.

**Objective:**

To evaluate maximal functional capacity in adults with isolated LBBB and compare it to healthy population‐derived predicted values (adjusted for sex, age, weight, and height).

**Methods:**

This descriptive pilot study included subjects with isolated LBBB derived from outpatient clinics of two academic hospitals. All subjects underwent maximal cardiopulmonary exercise testing (CPET) and a Global Physical Activity Questionnaire (GPAQ). The primary outcome was to evaluate maximal functional capacity according to population‐derived predicted values of peak oxygen consumption (peakVO_2_): pp‐peakVO_2_. The secondary outcome was to report adverse CV events (CV deaths or hospitalizations) at follow‐up.

**Results:**

A total of 27 (18 women and 9 men) participants were included. The median (interquartile range) age of the sample and time to screening from the first LBBB diagnosis were 62 (51−71) and 3.4 (1.1−8.4) years, respectively. The results of the GPAQ score showed that 19 patients were highly active, and 8 were moderately active. The median of peakVO_2_ and pp‐peakVO_2_ were 19.3 (15−22.5) ml/kg/min and 88% (79.3%−104.4%), respectively. There were no adverse CV events at a median follow‐up after CPET of 3.1 (2.7−3.4) years.

**Conclusion:**

In this pilot study, adults with isolated LBBB showed reduced maximal functional capacity, despite the absence of cardiac disease and a baseline moderate to highly active lifestyle.

## Graphical abstract

The effect of an isolated LBBB on maximal functional capacity. GPAQ, Global Physical Activity Questionnaire; LBBB, left bundle branch block.

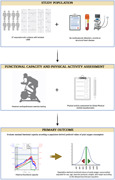

**Abbreviations**
CPET: cardiopulmonary exercise testing
CV: cardiovascular
LBBB: left bundle branch block
LVEF: left ventricular ejection fraction
PeakVO_2_
: peak oxygen consumption at maximal exercise
pp‐peakVO_2_
: percent predicted peak oxygen consumption at maximal exercise

## 1 INTRODUCTION

The prevalence of left bundle branch block (LBBB) in the absence of clinically detectable heart disease is not unusual (from 0.034% to 2.5%).^1^, ^2^, ^3^ Moreover, LBBB has been associated with adverse prognosis even in asymptomatic individuals without known cardiovascular (CV) disorders when LBBB has been an incidental finding on ECG.^4^ Although isolated LBBB has been reported to adversely affect left ventricular volumes and left ventricular ejection fraction (LVEF) in a small study,^5^ the effect of isolated LBBB on maximal functional capacity is unclear. Based on adverse prognosis findings from previous evidence in isolated LBBB^1^, ^2^, ^3^, ^4^ and the inverse association between functional capacity and prognosis in apparently healthy adults,^6^ we hypothesized that this disorder of cardiac ventricular conduction might be associated with lower functional capacity compared with the general population. Accordingly, this pilot study aimed to evaluate maximal functional capacity in adults with an isolated LBBB and compare it to population‐derived predicted values (adjusted for sex, age, weight, and height) of a healthy and sedentary population.

## 2 METHODS

This pilot study was a two‐center, prospective report. LBBB was defined according to current electrocardiographic (ECG) criteria.^7^ Research Ethics Committee approved the study protocol following the principles of the Declaration of Helsinki and national regulations. All patients who participated in the research provided informed consent.

The eligibility of candidate patients was based on the following inclusion criteria: (a) adult >18 years old, (b) ECG criteria of LBBB,^7^ and (c) provide informed consent. In addition, exclusion criteria were (a) inability to perform a maximal baseline exercise test; (b) structural heart disease, valve heart disease, or diastolic dysfunction estimated by two‐dimensional echocardiography; (c) previous ischemic heart disease, heart failure, myocardiopathy, or myocarditis; (d) previous history of known or suspected chronic coronary syndromes; (e) effort angina during cardiopulmonary exercise testing (CPET); (f) any pulmonary disease; (g) anemia, and (h) an LVEF < 55%.

Patients who met the inclusion−exclusion criteria and signed the informed consent form underwent a comprehensive medical history, physical examination, anthropometry, and examination tests. The examination tests included: an electrocardiogram (ECG), two‐dimensional transthoracic echocardiography, laboratory test, CPET, and baseline physical activity assessment by Global Physical Activity Questionnaire (GPAQ).

Maximal functional capacity was evaluated using incremental and symptom‐limited CPET on a bicycle ergometer, beginning with a workload of 10 W and increasing gradually in a ramp protocol at 10‐W increments every 1 min. We defined maximal functional capacity as when the patient stops pedaling because of symptoms and the respiratory exchange ratio (RER) was ≥1.1. During exercise, patients were monitored with 12‐lead electrocardiogram and blood pressure measurements every 2 min. Gas exchange data and cardiopulmonary variables were averages of values taken every 10 s. Peak oxygen consumption at maximal exercise (PeakVO_2_) was defined as the highest value of VO_2_ during the last 20 s of exercise. Once peakVO_2_ was obtained, we calculated its percent predicted peak oxygen consumption at maximal exercise (pp‐peakVO2), defined as the percentage of predicted peakVO_2_ adjusted for sex, age, exercise protocol, weight, and height according to the Wasserman/Hansen standard prediction equation for the healthy and sedentary population.^8^

The ventilatory efficiency was determined by measuring the slope of the linear relationship between minute ventilation (VE) and carbon dioxide production (VCO2) across the entire course of the exercise (VE/VCO_2_ slope).

The study's primary outcome was to evaluate maximal functional capacity according to population‐derived predicted values of peak oxygen consumption (peakVO_2_): pp‐peakVO_2_. The secondary outcome was reporting adverse CV events (CV deaths or hospitalizations) at follow‐up. Researchers blinded to the patient's baseline characteristics, including CPET parameters, informed the secondary outcome. All patients included were follow‐up until July 2022.

Continuous variables are expressed as means (±1 SD) or medians (interquartile range [IQR]), and discrete variables are as percentages.

## 3 RESULTS

A total of 27 consecutive asymptomatic adults with a previous diagnosis of LBBB recruited from general practitioner outpatient clinics were included in this pilot study. At baseline, the median age was 62 (51−71) years, 66.7% were women, 62.9% had a history of hypertension, and the median time from LBBB diagnosis to the screening visit was 3.4 (1.1−8.4) years. The results of the GPAQ score showed that 19 patients were highly active, and 8 were moderately active. All subjects screened reached the final study group. Participants' baseline characteristics are presented in Table 1.

**Table 1:** Baseline characteristics of participants

	(*n* = 27)
Demographic and clinical variables	
Age, years	62 (51−71)
Women, *n* (%)	18 (66.7)
BMI, kg/m^2^	27.7 ± 3.3
Hypertension, *n* (%)	17 (62.9)
Smoker, *n* (%)	2 (7.4)
Past smoker, *n* (%)	4 (14.8)
Dyslipidemia, *n* (%)	9 (33.3)
Diabetes, *n* (%)	4 (14.8)
QRS duration, ms	140 (130−160)
Echocardiographic parameters	
LVEF, %	59.3 (56−66)
TAPSE, mm	24 (21.2−28.8)
E/e' ratio	9.2 (7.6−11)
LV diastolic diameter, mm	49 (46−53)
LV systolic diameter, mm	31.5 (28.9−35)
Left atrial diameter mm	35 (32−37)
IVS thickness, mm	10.7 (9.6−11.5)
LV mass index, g/m^2^	99 (83−127)
CPET parameters	
HR at rest, bpm	78 (70−89)
HR at peak, bpm	153 (136−167)
SBP at rest, mm Hg	135 (125−140)
SBP at peak exercise, mm Hg	180 (160−190)
PeakVO2, ml/kg/min	19.3 (15−22.5)
PeakVO2, L/min	1.31 (1.04−1.88)
pp‐peakVO2, %	88 (79.3−104.4)
VE/VCO2 slope	28.1 (24.3−33.3)
Respiratory exchange ratio	1.25 (1.17−1.4)
Laboratory parameters	
Hemoglobin, mg/dl	13.8 (12.8−14.6)
NT‐proBNP, pg/ml	122 (55−195)
CA125, U/ml	9 (6−12)
eGFR, ml/min/1.73 m^2^	85.2 (75.3−106.6)

*Note*: Continuous variables are expressed as means (±1 SD) or medians (interquartile range [IQR]), and discrete variables as frequencies and percentages.

Abbreviations: BMI, body mass index; CPET, cardiopulmonary exercise testing; E/e', ratio between early mitral inflow velocity and mitral annular early diastolic velocity; eGFR, estimated glomerular filtration rate; IVS thickness, interventricular septum thickness; LV, left ventricular; LVEF, left ventricle ejection fraction; PeakVO2, peak oxygen consumption at maximal exercise; pp‐peakVO2, percent predicted peak oxygen consumption at maximal exercise; SBP, systolic blood pressure; TAPSE, tricuspid annular plane systolic excursion; VE/VCO2 slope, ventilatory efficiency.

### 3.1 Primary outcome

All participants performed a maximal CPET (RER > 1.1) limited by muscular fatigue. The median of peakVO_2_ was 19.3 (15−22.5) ml/kg/min. Compared with population‐derived predicted values of peak oxygen consumption (adjusting for sex, age, exercise protocol, weight, and height), the median of pp‐peakVO_2_ was 88% (79.3%−104.4%). Only seven subjects exhibit a pp‐peakVO_2_ > 100%. Of them, six subjects scored highly active in GPAQ and one moderately active. Thirteen participants exhibited a pp‐peakVO_2_ < 85%. Of them, nine subjects scored highly active in GPAQ and four moderately active.

### 3.2 Secondary outcome

At a median follow‐up after the screening visit of 3.1 (2.7−3.4) years and after the LBBB diagnosis of 7.1 (4.1−11.1) years, no adverse CV events (CV deaths or hospitalizations) were registered. In addition, none of the included patients was admitted for acute coronary syndrome or presented a suspected chronic coronary syndrome during follow‐up.

## 4 DISCUSSION

The main finding of this pilot study was that we observed a reduced aerobic capacity in a small sample of active adults with isolated LBBB compared with sedentary population‐derived predicted values.

Although previous literature has shown that isolated LBBB predicts adverse CV outcomes^1^, ^3^ and is associated with greater left ventricular volumes and reduced LVEF^5^ compared with the general population, the evidence remains scarce regarding LBBB effects on functional capacity in subjects without structural heart disease. Along this line, CPET, with incremental workload and symptom‐limited exercise testing, is considered the gold standard when studying maximal aerobic capacity in the general population.^8^ However, regarding physical performance in the LBBB population, only a previous cross‐sectional study conducted by Barros et al.^9^ evaluated the effects of this ECG conduction disturbance in 26 subjects with an LVEF > 50% and mainly a sedentary lifestyle. In congruence with our results, the authors reported a reduced baseline pp‐peakVO_2_ (87.2%) with a mean VE/VCO_2_ slope <30. However, after comparing subjects with LBBB to a nonmatched control group (23 subjects), the authors concluded that in the multivariate analysis (including covariates such as age, sex, and body mass index), LBBB was associated with VE/VCO_2_ slope and had no effect on pp‐peakVO_2_.

In the present work, we cannot unravel the underlying mechanisms by which LBBB could reduce exercise capacity in isolated LBBB. However, previous CV magnetic resonance evidence in subjects with isolated LBBB has postulated that electromechanical dissociation, rather than intrinsic myocardial abnormality, is a potential mechanism to explain the adverse effects of isolated LBBB on LVEF.^5^ Along this line, our results suggest that in the absence of a significant increase of biomarkers (such as natriuretic peptides or antigen carbohydrate 125) or CPET parameters (such as VE/VCO_2_ slope), a congestive mechanism should not be the principal contributor explaining the present findings. Nevertheless, the present data should be interpreted as a “proof of concept” idea. Further studies, including carefully selected control‐matched groups and cardiac stress imaging testing, must confirm these results and elucidate the underlying pathophysiological mechanisms responsible for these effects.

Several limitations need to be acknowledged. First, this pilot study has the inherent limitations of being a work with a relatively small number of participants, a fact that may limit the extrapolation of these findings to the entire population of individuals with isolated LBBB. Second, we did not include a control‐matched group. Third, oxygen pulse was not evaluated. Fourth, we did not perform cardiac stress imaging or invasive tests to exclude ischemic heart disease. Finally, with the current data, we cannot unravel the pathophysiological mechanism behind these findings.

## 5 CONCLUSIONS

In this pilot study, adults with isolated LBBB showed reduced maximal functional capacity, despite the absence of structural heart disease and a baseline moderate to highly active lifestyle. Further studies must confirm these results and elucidate the underlying pathophysiological mechanisms responsible for these effects and the potential prognostic impact at long‐term follow‐up.

## CONFLICT OF INTEREST STATEMENT

The authors declare no conflict of interest.

## Data Availability

The data supporting this study's findings are available from the corresponding author upon reasonable request.

## References

[1] Fahy GJ , Pinski SL , Miller DP , et al. Natural history of isolated bundle branch block. Am J Cardiol. 1996;77(14):1185‐1190. 10.1016/s0002-9149(96)00160-9 8651093

[2] Hardarson T , Árnason A , Elíasson GJ , Pálsson K , Eyjólfsson K , Sigfússon N . Left bundle branch block: prevalence, incidence, follow‐up and outcome. Eur Heart J. 1987;(10):1075‐1079. 10.1093/oxfordjournals.eurheartj.a062172 3678236

[3] Azadani PN , Soleimanirahbar A , Marcus GM , et al. asymptomatic left bundle branch block predicts new‐onset congestive heart failure and death from cardiovascular diseases. Cardiol Res. 2012;(6):258‐263. 10.4021/cr214w 28352414 PMC5358299

[4] Surkova E , Badano LP , Bellu R , et al. Left bundle branch block: from cardiac mechanics to clinical and diagnostic challenges. EP Europace. 2017; 19(8):1251‐1271. 10.1093/europace/eux061 28444180

[5] Akhtari S , Chuang ML , Salton CJ , et al. Effect of isolated left bundle‐branch block on biventricular volumes and ejection fraction: a cardiovascular magnetic resonance assessment. J Cardiovasc Magn Reson. 2018;20(1):66. 10.1186/s12968-018-0457-8 30231875 PMC6146610

[6] Imboden MT , Harber MP , Whaley MH , Finch WH , Bishop DL , Kaminsky LA . cardiorespiratory fitness and mortality in healthy men and women. JACC. 2018;72(19):2283‐2292. 10.1016/j.jacc.2018.08.2166 30384883

[7] Strauss DG , Selvester RH , Wagner GS . Defining left bundle branch block in the era of cardiac resynchronization therapy. Am J Cardiol. 2011;107(6):927‐934. 10.1016/j.amjcard.2010.11.010 21376930

[8] Wasserman K . Principles of Exercise Testing and Interpretation: Including Pathophysiology and Clinical Applications. Lippincott Williams & Wilkins; 2005:160‐182.

[9] Barros MS , Amorim RS , Rocha RO , et al. Cardiopulmonary exercise testing in patients with left bundle branch block and preserved ejection fraction. In. J Cardiovasc Sci. 2017;30:11‐19. 10.5935/2359-4802.20170018

